# The Adoption of Bundled Sustainable Farm and Environmental Practices by Coffee Farmers in Southwest Ethiopia

**DOI:** 10.1155/2021/9954230

**Published:** 2021-12-08

**Authors:** Gezahagn Kudama, Hika Wana, Mabiratu Dangia

**Affiliations:** ^1^Wollega University, Shambu Campus, Department of Agricultural Economics, Shambu, Ethiopia; ^2^Gambella University, Department of Agricultural Economics, Gambella, Ethiopia

## Abstract

Despite numerous efforts to introduce sustainable farm and environmental practices (SFEPs), such as pruning, soil erosion control, and water pollution abatement measures), their adoption by smallholder farmers is awfully low in Ethiopia. As a result, smallholder coffee farmers in the country remain in poverty traps even if there is room to enjoy coffee returns by doubling the yield by implementing sustainable practices. On the other hand, most previous coffee sustainability studies focus on the economic, livelihood, and poverty alleviation impact of private sustainability standard schemes. Despite the holistic advantages of the adoption of bundled SFEPs over individual adoption practices, it has been overlooked by earlier scholars in the country. In southwest Ethiopia, few farmers applied sustainable coffee farm practices (particularly pruning, stumping, the use of fertilizer, and mulching), and the yields gained by the farmers are quite low. Therefore, this study seeks to examine the factors affecting the adoption of bundled SFEPs and their intensity at the farm household level in southwest Ethiopia based on cross-sectional data obtained from 153 sampled coffee farm households for the 2019/2020 cropping season. The study results showed that the farmers' adoption of different SFEPs depended on farm and management characteristics (total size of coffee holdings, multiple plots, remoteness of coffee farm, hired labor, and farming experience), socioeconomic variables (literacy, household size, and training), and Fairtrade coffee certification. Likewise, the intensity of SFEPs implementation is influenced by literacy and hired labor. Providing training and supplementing coffee farmers with farm equipment used for SFEPs, promoting small-scale mechanization options to address seasonal labor constraints, as well as strengthening Fairtrade organizations will facilitate the adoption of multiple SFEPs by coffee farmers in the country.

## 1. Introduction

Ethiopia is the origin of Arabica coffee [1] and the biggest producer and exporter in Africa [2]. Coffee is one of the uppermost contributors to the Ethiopian government's capital through taxation, social services, and trade, [3] accounting for 34% of the total national export earnings in 2017/18 [4]. The sector provides an income basis for an estimated 15 million Ethiopians [1]. Ethiopia has also a specific coffee consumption culture, and the domestic consumption is estimated to be 50% of the national production [3, 5].

All coffee production is rain-fed in Ethiopia, and 95% of national production is contributed by smallholder farmers who grow their coffee in different environments, including forests, semiforests, gardens, and plantations [4]. Coffee production in Ethiopia is largely organic [6], although tremendously few farmers use chemicals. 6% of coffee producers are estimated to be using chemical fertilizer and 2% use other agrochemicals [6].

From a development viewpoint, although improvements (such as coffee farm management and soil conservation practices) have been made to coffee, productivity is still low [4] compared to major coffee producer countries like Vietnam and Brazil. As per the USAID [7] data, with a mean of 0.75 tons per hectare of land, Ethiopia has nearly 50% and 70% lower coffee yields than Vietnam and Brazil, respectively. By the same token, approximately 5 million smallholder coffee farmers throughout East Africa have 50% lesser coffee yields than those in Central America, largely because of the lack of the adoption of good coffee farm management practices [8].

On the other hand, most coffee-producing regions suffer from high levels of poverty incidence in the country. The study result conducted on rural poverty and sustainability indicators in coffee-growing regions of south and southwestern Ethiopia shows that an estimated 65–70% of farmers in the targeted coffee regions are below the US$3.10 per day poverty line [9]. This is much higher than the rate of poverty at the national level that is estimated to be 23.5% in 2015-16 [10]. The high level of poverty might be associated with a low level of SFEPs adoption status in the area. Coffee production sustainability scores (environmental, social, and economic standards) are largely correlated with poverty levels. In other words, wealthier farmers tend to have better sustainability scores in general than poorer farmers [9].

To shift the existing conditions of the coffee sector, numerous encouraging reforms are under implementation. Particularly, the Ethiopian government's second Growth and Transformation Plan involves increasing coffee yield and production. However, the performance of the sector has failed to meet the expectations, pointing out that the government policy fails to consider the real situation on the ground—the prevailing bad coffee tree management measures and limited supply of modern inputs [4]. The key barriers to meeting these goals are the main factors related to poor farm management practices and improved technology adoption. Although there is room to more than double the coffee yield and net income of coffee with good agricultural practices and rejuvenation, the adoption of sustainable farms and technology is awfully low in Ethiopia. For instance, approximately 80% of the total coffee land needs rehabilitation and renovation practices in Ethiopia to fully meet its potential coffee yield improvement. On the other hand, based on the global coffee platform and Technoserve estimation, the country has the potential to uplift smallholder coffee yield up to 114%, with renovation, rehabilitation, and other sustainable good agricultural practice in the country [7]. As a result, the sustainability of coffee growing has a valuable effect on the poverty alleviation and livelihoods of smallholder coffee farmers in Ethiopia.

The thought of sustainable practices has been extensively encouraged in agriculture. It is crucial to realize that the current resource use may exhaust to meet the future generation demand for resources continuously. Sustainable agricultural improvement can be realized by the uptake of either particular or multiple sustainable agricultural practices. The adoption of individual sustainable practices is often used to help a specific target. For example, cutoff drains and mulches are commonly used to control soil erosion. On the other hand, bundled sustainable agricultural practices are the combinations of different individual sustainable practices. For instance, Good Agricultural Practice (GAP) and organic certification schemes include different sustainable agricultural practices for soil and water conservation, soil fertility management, pest management, and waste management. Moreover, the bundled sustainable agricultural practices are more of an all-inclusive approach since they comprise a set of multifunctional measures that advance agricultural sustainability in general [11]. Despite several benefits of bundled sustainable practices, their adoption rates are exclusively low in the sub-Saharan Africa [12–15].

Across different kinds of literature, the socioeconomic, institutional, environmental, and climatic features affecting the adoption of bundled sustainable agricultural practices throughout the sub-Saharan Africa have been explored in different contexts. It justifies that the adoption variables differ in terms of household characters considered, technology, and location [14]. Furthermore, the findings of different study results on the adoption packages of sustainable agricultural practices are not clear yet [11]. Several studies have explored the approaches followed by different voluntary sustainability standards (VSS) and their effects on coffee sector sustainability in Ethiopia [16]. However, most studies have investigated the economic, livelihood, and poverty alleviation impact of smallholder participation in private sustainability standard schemes [5, 17]. In southwest Ethiopia, few farmers applied sustainable coffee farm practices (particularly pruning, stumping, the use of fertilizer, and mulching), and the yields gained by the farmers are quite low. For example, the average coffee yield was 350 kg green beans per hectare for farmers, while there is a possibility to produce 1,000 kg green beans on the same unit of the farm for well-managed semiforest coffee farms with improved varieties in the area [18].

The present study aims at examining the factors affecting the bundled adoption of sustainable farm and environmental practices (SFEPs) among coffee farm households in southwest Ethiopia. The study adds value to the existing literature. Firstly, this study offers the first attempt to explicitly evaluate the drivers of the adoption of bundled SFEPs for the coffee sector in Ethiopia. Secondly, this study identifies various SFEPs choices adopted by coffee farm households while distinguishing the linkage between the different SFEPs considered. Hence, the findings in this study will help in the design of more effective policy tools for promoting coffee-related technology adoption. In particular, this study seeks to examine the following research questions: (1) what are the factors affecting the adoption of bundled SFEPs in the coffee sector at the farm household level? (2) What are the factors influencing the adoption intensity of SFEPs?

## 2. Review of Empirical Studies

Sustainable agricultural practices are the measures that help improve farming productivity with small negative effects on the environment. However, the adoption of these measures by smallholder farmers has exclusively been slow in most Sub-Saharan Africa [19]. The adoption of bundled sustainable agricultural practices is unavoidable since the practices are used frequently in combination [15]. The adoption of bundled sustainable practices is not uniform, where several intrinsic and extrinsic factors determine individual adoption performance [15, 20]. Furthermore, farmers do not necessarily implement the recommended SAPs blindly as the adoptive decision-making faced by the farmers relies on a mix and match of SAPs with similarity to local conditions [11]. The interactions of different features influencing the farmers' decision to adopt different practices are explained below based on the past studies' findings as related to this study.

Among the farm and management characteristics, the farm size is an important factor driving or constraining the adoption of sustainable farm practices. A large farm size facilitates the adoption of sustainable farm practices since farmers who possess large-sized farms have more capacity to allot resources and distribute the risk across larger areas [11, 21]. In contrast, a farmer with a large farm size might prefer extensive farming to intensive farming [19, 22, 23]. Operating on many farms can enhance the adoption of multiple sustainable farms and environmental management practices since the situations on different plots may seek the adoption of different practices [24]. On the other hand, the study results by Sileshi et al. [25] showed the probability of farmers adopting bench terracing decrease with an increase in the number of plots. Access to hired labor is also a crucial input for facilitating the uptake of multiple SAPs by overcoming labor force supply limitations for adoption practices [23]. On the other hand, the average remoteness of coffee farms from home to farm is less likely to influence the adoption of multiple sustainable practices by farmers because of the transportation costs of materials and the opportunity cost time involved in transporting different materials from remote [26]. A large household size, which is a proxy to household labor resources, may enhance the likelihood of farmers' adoption decisions of bundled sustainable farm practices since most of the practices are labor demanding activities [19].

Studies have also unmasked the socioeconomic characteristics of the farm households (farmers' education, age, farming experience, training, and information variables are indicators) as reliable indicators of the predisposition to adopt sustainable farm practices [11]. Among such possible signals, the direction of the effect of age and education is not always clear [20]. In several adoption works of literature, age, which is a proxy to farming experience, is an important influencing factor in new agricultural technology adoption. However, the effect of age is not uniform with the adoption of multiple sustainable farm practices [20, 23]. Education is supposed to enlighten the household head on the adoption of bundled sustainable farm practices and make it easy to understand the benefits of sustainable farm practices. However, the effects of education on the household showed mixed results on the adoption decisions with differing perspectives [23]. Similarly, the receipt of training on the sustainable farm and environmental practices helps to enlighten the farmers on the importance of sustainable practices. Likewise, the study conducted by Nigussie et al. [23] showed that participation in training on sustainable practices helps widen the understanding of the importance of manure to soil fertility and then increases their use of manure.

From a social capital perspective, the cooperatives may encourage adoption by creating social networking and conducive environments for farmers to share their knowledge, know-hows, and experiences about sustainable practices [19, 20]. Finally, the membership of Fairtrade encourages the adoption of sustainable farm and environmental practices. Voluntary sustainability standards (VSS), such as Fairtrade and organic certification schemes, have scaled up in Brazil's coffee sector [27].

## 3. Method

### 3.1. Description of the Study Area and Sampling Procedure

The data for this study was collected from 153 coffee farm households in the Jimma Zone, Oromia National regional state in southwest Ethiopia. The survey related the activities undertaken during the 2019/2020 coffee season and was conducted in October 2020. Field interviews and face-to-face interviews with the household heads were conducted using a structured questionnaire. During the initial sampling, the Jimma zone was selected because of its high potential for coffee production among coffee-growing areas within the country, where 30–45% of the population of the zone relies on coffee production [17].

In this study, the sample size was determined based on a simplified formula developed by Yamane [28] at 0.081 sampling error. The formula is specified as,

(1) $$\begin{array}{c} n=\frac{N}{1+N{\left(e\right)}^{2}}, \end{array}$$

where *n* is the sample size, *N* is the population size (the total smallholder coffee producer households of Jimma zone), and *e* is the level of precision. Based on CSA [29] data, Jimma zone had a total of 504,336 smallholder coffee producer households in the 2019 cropping season. Hence, the desired sample size is equal to 153 households.

In the second stage, two districts (Gera and Seka Chekorsa) within the zone were randomly selected as field sites, and the survey was pretested in each district. Proportionate random sampling was conducted using a database of coffee producers obtained from the respective agricultural and rural development offices of each district.

### 3.2. Sustainable Farm and Environmental Practice Categories

The data that includes all dimensions of sustainability attributes based on an international and established sustainability framework hardly exists for the coffee sector [16]. Moreover, the sustainability standards in the coffee sector vary from organization to organization.

On the other hand, a set of sustainability standards introduced by Technoserve under its Coffee Initiative project is commonly used in East Africa. The standards obey universally accepted environmental, social, and safety-related best practices, categorized into five broad sustainability practices: social, health and safety, environmental, economic, and farm management [30]. In this study, following Technoserve's sustainability classifications, due attention is given merely to sustainable environmental and farm management practices to meet the objective of the study. Furthermore, the classification set by Technoserve is too wide to include under one category. It seeks a further subdivision based on their main contribution to sustainability in the coffee sector. Accordingly, the farm management practices are further categorized into the renovating coffee tree stock practices that allow the coffee trees to bear more and the application of fertilizer practices that improve plant nutrition. Similarly, the environmental practices are classified into the soil conservation practices that permit erosion control and the water environment management practices used for water pollution control. Consequently, the four main categories of sustainable farm and environmental practices (SFEPs), which are common among coffee farm households in Ethiopia, are considered in this study. They are as follows:

#### 3.2.1. Renovating Coffee Tree Stock

- Replanting coffee trees
- Pruning coffee branch
- Stamping coffee stock

#### 3.2.2. Applying Fertilizer

- Organic fertilizer usage
- Chemical fertilizer usage

#### 3.2.3. Implementing Soil Conservation Practices

- Constructing terraces
- Cutoff drains
- Mulching
- Intercropping with food crops

#### 3.2.4. Water Environment Management Measures

- Dumping waste from coffee processing 30 meters or more away from water sources
- Using a latrine 30 meters or more away from water sources
- Using washing places 30 meters or more away from water sources

For each of the four categories of sustainable management practices and water pollution abatement measures, a farmer was considered an adopter if at least one of the practices had been applied. The first category lists three possible options the producers have for improving coffee tree stock. These measures embrace replanting the plots by removing the old or damaged trees, pruning the branches, or cutting off the trunks (stumping) to inspire new growth. Naturally, it takes three years for the coffee trees after planting to begin cherries with peak production ranging from five to six years. After a peak period, the coffee yields begin to decline unless it gets pruned or stumped and the type of measure it requires rests on both the physical state and age of the tree [20]. For the second category of sustainable management practices, organic and chemical fertilizer utilizations were considered although Ethiopian coffee farmers hardly use chemical fertilizers [6]. The types of organic fertilizers used in coffee production include manure, coffee pulp, and compost.

The third category of sustainable management practices summarizes the ways the farmers can reduce soil erosion because of water runoff. Such practices involve constructing terraces, cutoff drains, mulching, and intercropping with other food crops. Conservation measures are vital since coffee is mostly grown in mountainous areas, where coffee farms are often susceptible to soil erosion in the country.

The fourth category focuses on water pollution abatement measures. These measures include dumping waste from coffee processing, using a latrine, and washing places 30 meters or more away from the nearest water bodies. Water pollution abatement adoption measures are important since dumping waste from coffee processing into water sources is a serious problem in the coffee-growing regions of Ethiopia. Water quality worsens in the coffee-growing areas in Ethiopia because of the dumping of coffee waste into the rivers [31].

### 3.3. Econometric Model Framework

#### 3.3.1. The Multivariate Probit Model

The Multivariate Probit (MVP) model simultaneously estimates the effect of the set of explanatory variables on each of the various adoption practices, allowing the possible relationship among a farmer's decision to adopt one practice with other adoption choices, as well as the correlation between the unobserved error terms. On the other hand, several kinds of literature employ the univariate models to test the variables affecting the adoption of a sole practice. However, such a method may be problematic as it is incapable to consider the intercorrelations among the practices [15, 20]. Such correlations allow the error terms for positive correlation (complementarity) and negative correlation (substitutability) between multiple adoption measures [32].

Following Teklewold, Kassie, and Shiferaw, as well as [15] Ubertino, Mundler, and Tamini, [20] a random utility function of an *i*^th^ coffee farm household confronted with a decision to adopt or not adopt a bundle of interreliant sustainable management and water pollution control practices was considered. The utility *U*_*a*_ represents the benefits derived by the households from adopting the traditional agricultural practices, and *U*_*n*_ represents the benefits of adopting the sustainable management and water pollution control practices, which, in this context, include the adoption of renovating coffee tree stocks, fertilizer, soil conservation, and water pollution control practices. The farm household chooses to adopt if *Y*_in_^*∗*^=*U*_*n*_ − *U*_*a*_ > 0. The net benefit (*Y*_in_^*∗*^) from adoption is a latent variable determined by a vector of the farm household and characteristics (*X*_in_), as well as nonobservable disturbances captured by the error term *ε*_in_. This is specified as follows:

(2) $$\begin{array}{c} Y_{\text{in}}^{*}=\beta_{n}\cdot X_{\text{in}}+\varepsilon_{\text{in}}, \end{array}$$

where *β*_*n*_ represents the vector of parameters to be estimated. The observed binary outcome equation for each choice of practice adopted by the farm households is illustrated as follows:

(3) $$\begin{array}{c} Y_{\text{in}}=\left\{\begin{array}{c} 1,\;\text{ if }Y_{\text{in}}^{*}>0,\\ 0,\;\text{ otherwise.} \end{array}\right. \end{array}$$

If the adoption of multiple practices is assumed to be interdependent or is assumed to occur simultaneously, then the error terms in equation (2) are expected to jointly follow a multivariate normal distribution pattern with a zero conditional mean and a unitary variance [32, 33]. Hence, equation (3) displays a multivariate probit model that represents the decision to adopt multiple sustainable management and water pollution control adaptation practices, simultaneously.

#### 3.3.2. Ordered Probit Model

The MVP model from the previous subsection conceptualizes that a farm household will adopt new sustainable practices if the expected net benefit of the practices exceeds nonadoption. However, the MVP model cannot compute the intensity of the adoption of sustainable practices [14].

Adoption intensity is commonly estimated based on the proportion of the area of total cultivated land under a given practice but the exact area under each sustainable practice is hardly accessed [34]. However, under such data limitation, the intensity of adoption can be estimated using an ordered probit model by taking the number of sustainable practices adopted by each farm household as the dependent variable [14]. In this study, an integer ranging from 0 to 4 is assigned for the number of sustainable practices adopted by a farm household. Following Aryal et al. [34] and Teklewold et al. [15], the ordered probit model is illustrated as follows:

(4) $$\begin{array}{c} Y_{i}^{*}=X_{i}^{\prime }\beta +\mu_{i}, \end{array}$$

where *Y*_*i*_^*∗*^ is an underlying unobserved measure of the households' adoption of sustainable practices in numbers and is given by,

(5) $$\begin{array}{c} \left\{\begin{array}{c} \cdot \\ \cdot \\ Y=\;0,\;\text{if }Y^{*}\;\leq 0,\\ Y=1,\;\text{if }0\;<Y^{*}\leq \alpha_{1},\\ Y=2,\;\text{if }\alpha_{1}\;<Y^{*}\leq \;\alpha_{2},\\ \cdot \\ \cdot \\ \cdot \\ Y=4,\;\text{if }\alpha_{3}\;\leq Y^{*}, \end{array}\right. \end{array}$$

where the values of *y* are latent variables and *α* are unknown parameters to be estimated. For *m* categories following a standard ordered probability model, the probability of observing the outcome *i* corresponds to,

(6) $$\begin{array}{c} \mathrm{Pr}\left(\text{outcome}{\;}_{j}=i\right)=\mathrm{Pr}\left(K_{i-1}<X_{i}^{\prime }\beta +\mu_{i}\leq \alpha_{i}\right., \end{array}$$

where *μ*_*i*_ is assumed to be normally distributed with a standard normal cumulative distribution function. The coefficients *β*_1_ … *β*_*K*_ is jointly estimated with the cut points *α*_1_, *α*_2_ … *α*_*K*−1_, where K is the number of possible outcomes.

#### 3.3.3. Explanatory Variables

The dependent and explanatory variables encompassed in the model are discussed below.  Table 1 presents the definitions and descriptive statistics of the variables.

## 4. Results and Discussion

### 4.1. Descriptive Statistics

The survey data indicate that more than half of the responses were nonadopted for all categories of sustainable management and water pollution abatement practices, while the adoption rate varies substantially from one category to the other. For example, the number of producers who had adopted water pollution abatement practices was relatively high: 49% of the respondents had practiced at least one of the water pollution reduction practices. In contrast, only 24% of the farmers had implemented at least one measure of the soil conservation strategies. Concerning fertilizer application, 33% of the producers had applied fertilizer, and with three exceptions, all adopters had used organic materials. 74% of the farmers had renovated their coffee tree stocks. Based on the recent findings of related studies, the independent variables assumed to affect the dependent variables are described in Table 1.

### 4.2. General Performance of Multivariate Probit Model

The multivariate probit (MVP) model was used to estimate the effects of explanatory variables on the adoption behavior of coffee farmers. The log of some continuous variables was used for a better model fit. Before computing the model results, the existence of multicollinearity was checked by running the variance inflation factor (VIF). The mean results of the test (VIF = 1.69) indicate the nonappearance of a serious multicollinearity problem in the model. The Wald test [chi − square(40)=134.96^*∗∗∗*^] also designates that the data fit of the model performs reasonably well. Furthermore, a likelihood ratio test was carried out to compare the four univariate probit models with the multivariate results (Table 2). The test result was statistically meaningful [chi − square(6)=47.98^*∗∗∗*^], indicating that the error correlations between the farm management practices are jointly significant. It suggests that using the MVP model is preferable than individual probit regressions and supports the hypothesis that sustainable farm management adoption choices are interrelated. More specifically, the coffee farmers who practiced water management were more likely to renovate their coffee tree stock (rho=0.485^*∗∗∗*^), apply fertilizer (rho=0.763^*∗∗∗*^), adopt soil conservation practices (rho=0.717^*∗∗∗*^), practice water management (rho=0.660^*∗∗∗*^), and renovate coffee tree stock (rho=0.326^*∗∗*^). Similarly, the farmers who adopted soil conservation practices were more likely to apply organic fertilizers (rho=0.422^*∗∗∗*^). However, renovating the coffee tree stocks was not correlated with applying organic fertilizers and soil conservation practices.

#### 4.2.1. Farm Characteristics and Management Practices

Table 3 presents the MVP results obtained for the four categories of sustainable farm management practices. The result shows that the larger coffee producers are less likely to adopt soil conservation practices. This preference is likely because soil conservation measures are tedious and labor-demanding activities that entail high labor for the larger coffee farm sizes. Consequently, adopting soil conservation practices will be more attractive to the smaller producers since the practices require a higher amount of labor than what the larger producers can do. The finding is concurrent to that of Mulinde et al. [21] and Sileshi et al. [25].

The results indicate that multiple plots are negatively correlated with the application of fertilizer. With three exceptions, the interviewed farmers who adopted fertilizer had chosen to use organic fertilizer. However, this result is not unusual given that the coffee producers in Ethiopia have a habit of not applying chemical fertilizers. Tremendously, few farmers use chemicals (chemical fertilizers, pesticides, and herbicides) for growing coffee in Ethiopia, and most coffee production is organic [6]. For example, only 24% of coffee growers applied compost to their coffee farms in 2014 in Ethiopia [6]. Coffee producers who have multiple plots are less likely to use fertilizer. It is because farmers may disguise distributing organic fertilizer over several plots since the opportunity cost of time and transaction cost will be higher for multiple plots. The finding is concurrent to that of [25].

The MVP results indicate that coffee farming experience is a determining factor although the effect is not uniform. Experienced producers are more likely to apply fertilizer than their stock of coffee trees. This preference is likely because the experienced producers on an average have accumulated a stock of information on the benefits of fertilizer application. Similar to the previous study, i.e., Jabbar et al. [35], the study results have revealed a positive and significant relationship between the farming experience and the adoption of organic manure. In contrast, the coffee farming experiences, a proxy to age, were found to have a negative and significant relationship with the adoption of water management practices, indicating that experienced farmers/older farmers are less likely to adopt water management practices. This can be justified as age is commonly related to reduced physical capacities, however, many soil and water conservation measures are labor intensive [36]. It makes the experienced farmers selective among multiple sustainable practices, i.e., the water management practice decisions of these farmers would rely more on coffee farm production and profitability than on sustainable water management practices considerations. Consequently, they may not be interested in investing their physical resources and time in water management practices and focus on practices that provide them with a higher yield and profit.

The results designate that the remoteness of a coffee farm negatively influences the adoption of water management practices. The possible explanation for it is that the farmers who live far away from the plots may limit the amount and number of water management practices they choose to implement since these measures comprise the building of latrines and preparing a solid and liquid waste dump. Furthermore, implementing these measures entails higher transportation costs involved in moving heavy materials and opportunity costs of time.

The result also indicated that the farmers who use hired labor are more likely to adopt soil conservation and water management practices. It is plausible because access to hired labor may have the potential to facilitate the adoption of soil conservation and water management practices by enabling the farmers to overcome labor force-related production limitations.

#### 4.2.2. Farmers' Socio-Economic Character

The results indicate that literate farmers are less likely to renovate their stock of coffee trees and adopt water management practices. Although education is commonly believed to help farmers to adopt multiple sustainable farm and environmental practices (such as water pollution control measures), several of the water management and renovating coffee tree recommendations are labor and capital intensive. On the other hand, the opportunity costs of applying these practices will be larger for literate farmers. Hence, literate farmers may have better opportunities for off-farm activities and pay less attention to farm practices. In contrast with a previous study of Arslan et al. [37], this study's results have revealed a negative and significant relationship between literacy and the adoption of coffee tree stock renovation and water management measures, partially in line with the findings of Nigussie et al. [23] and Ubertino et al. [20].

The result showed that the family size is an influential factor, although the effect is mixed. The study result indicated that the farmers with a large family size are more likely to renovate their coffee stock. In contrast, the findings reveal that the families with a larger number of people are less likely to adopt water management practices.

Similar to the former, several works of literature often suggest that the family size has a positive influence on the adoption of labor-intensive related practices since it is considered a proxy to family labor supplies [32]. However, this may not be the case for environmental sustainability practices since the influence of several environmental management measures (such as water pollution control) will not be detected overnight. Similarly, water quality management measures, such as stream fencing, are less preferred as their benefits on water quality take a long period to be observed [38]. Likewise, coffee farmers in the study area seemed to give higher emphasis to practices that maximize economic returns in a short period than environmental sustainability. A similar result was found in Mexico where coffee farm households who owned large family sizes were less likely to adopt soil conservation practices [20].

The results also reveal that the farmers who have participated in the coffee farm management training are more likely to use fertilizers in their coffee production. It is because training may have a positive effect on the accumulation of coffee-related knowledge by the farmers, which encourages them to use improved inputs (such as compost). The explanation of the finding was in agreement with the study results by Nigussie et al. [23], which revealed a positive and significant relationship between the sustainable land management training receipts by the farm households and the adoption of organic manure in northwestern Ethiopia.

#### 4.2.3. Fairtrade Coffee Certification

Finally, the estimates disclose that the farmers who have affiliation with Fairtrade are more likely to renovate their coffee tree stock and adopt water management practices because the Fairtrade scheme highly encourages the coffee-related technology adoption (such as coffee pruning and stumping) in line with environmental sustainability (such as water pollution control) in the study area. Hence, the certification of voluntary sustainability standards such as Fairtrade certification increases, significantly, the adoption of improved coffee practices in Ethiopia [39]. Likewise, the farmers with Fairtrade coffee certification are more likely to adopt environmental sustainability measures that involve water management practices, which is in line with the findings of Giuliani et al [40].

### 4.3. Ordered Probit Model Results

Table 4 presents the results of the ordered probit model of the factors influencing the number of SFEPs adopted among the sampled coffee farm households. The chi-squared statistics from the order probit model are statistically significant (*P* < 0.001) (chi2(10) = 46.69, Prob > chi2 < 0.001), designating that the model is of good fit. The results further show that only literacy and hired labor were found to be the significant factors influencing the number of SFEPs adoption decisions of the coffee farm households among other variables used in the model.

On the other hand, the results indicate that the literacy of the household head is likely to influence the adoption of the number of SFEPs. Although education is commonly considered helpful for farmers in adopting SFEPs, literate farmers often have better opportunities for off-farm activities and may limit the number of practices they choose to implement. The finding of this study is partially in agreement with the study results of Nigussie et al. [23], which indicated that the literacy level of small-scale farmers is supposed to have a negative influence on the adoption of many sustainable land management practices (stone-faced soil bund, traditional stone bund, and inorganic fertilizer technologies) in Ethiopia.

In terms of hired labor, the results of the study suggest that the farmers who employ hired labor are more likely to implement the number of SFEPs. The farmers are more likely to adopt different sustainable land management practices with the aid of hiring off-farm labor, however, they are less likely to adopt [23]. Similarly, the study results confirm that hired labor facilitates the intensity of adoption.

## 5. Conclusions and Policy Implications

In East Africa, coffee farming is characterized by poor productivity, largely because of the low adoption of best sustainable practices by the smallholder coffee farmers. Furthermore, compared with other East African countries (Kenya and Rwanda), the adoption of best sustainable practices is very low in Ethiopia. As a result, the smallholder coffee farmers in the country remain in poverty traps although the country has a high potential to increase the coffee production and returns at large by implementing multiple sustainable practices. On the other hand, the bundled sustainable coffee farms and environmental practices seem overlooked by scholars since most previous sustainability studies focus on the economic, livelihood, and poverty alleviation impact of smallholder participation in private sustainability standard schemes. Therefore, the present study examined the factors affecting the adoption of bundled sustainable farm and environmental practices (SFEPs) and their intensity at the farm household level in southwest Ethiopia based on cross-sectional data obtained from 153 sampled coffee farm households for the 2019/2020 cropping season.

The study results showed that the farmers' adoption of different SFEPs and their intensity of implementation depends on farm and management characteristics (total size of coffee holdings, multiple plots, remoteness of coffee farm, hired labor, and farming experience), socioeconomic variables (literacy, household size, and training), and holding Fairtrade coffee certification.

In particular, the study results reveal that the adoption of renovated coffee tree stocks was positively influenced by family size and Fairtrade certification holding, while it was influenced negatively by the literacy of the head of the household. Similarly, the adoption of organic fertilizer was positively influenced by coffee farming experience and training receipt of farmers, while it was influenced negatively by multiple coffee plots. Likewise, the implementation of soil conservation measures was positively influenced by hired labor, while it was influenced negatively by coffee farm size. The implementation of water management practices was positively influenced by hired labor and Fairtrade certification holding, while it was influenced negatively by the remoteness of the coffee farms, family size, coffee farming experience, and literacy of the head of the household.

Finally, the farmer decisions to adopt the number of SFEPs were positively affected by hired labor, while they were affected negatively by the literacy of the head of the household.

In general, the adoption of different SFEPs varies with the farm and household characteristics. As a result, the policy measures should vary for specific farm and household characteristics to promote SFEPs. For example, the measuring supports of coffee technology-related practices such as coffee tree stock renovation should be prioritized for literate small family size coffee farmers, whereas the use of organic fertilizer treatments should be promoted to the inexperienced (younger) and multiple coffee farm holders. Likewise, the sustainable development measure that supports soil conservation practices should be prioritized for a larger coffee farm householder. Similarly, the sustainable environmental measure supports of water quality management practices should be prioritized for the experienced (older), literate, larger householding coffee farmers. Hence, this might be promoted by training, awareness creation concerning sustainable farm management, environmental practices, and asset-building interventions.

The adoption of SFEPs measures was also affected by the receipt of training, hired labor, and Fairtrade coffee certification. Additionally, hired labor and literacy of the head of the farm households were also significant predictors of the intensity of bundled SFEPs that are adopted by coffee farmers. Farmers should be encouraged by providing training and supplementing them with farm equipment (used for coffee stock renovation, physical soil and water conservation measures, and toilet construction), promoting small-scale mechanization options to address seasonal labor constraints, as well as the strengthening of Fairtrade or related VSS organizations to induce learning-from-peers effects to facilitate the adoption of multiple SFEPs by coffee farmers in the country.

## Figures and Tables

**Table 1:** Definitions of variables and descriptive statistics.

Dependent variables	Name	Mean	Std.Dev.

Renovating coffee tree stock (1 = yes; 0 = no)		0.41	0.49
Applying fertilizer (1 = yes; 0 = no)		0.33	0.47
Implementing soil conservation practices (1 = yes; 0 = no)		0.24	0.43
Practicing water management (1 = yes; 0 = no)		0.49	0.5
Explanatory variables			

Farm and management characteristic			
Coffee farm size (hectare)	Farsize	0.57	0.50
Farmer has multiple plots (1 = yes; 0 = no)	Multplot	0.84	0.36
Average remoteness of coffee farm from home (minutes)	Remot	14.41	13.06
Farmer uses hired labor (1 = yes; 0 = no)	Hiredlab	0.08	0.27
Coffee farming experience of farmer (years)	Exper	11.88	5.11
Socio-economic characteristic			
Literacy status of farmer (1 = bread and write; 0 = does not bread and write)	Literacy	0.59	0.49
Family size (number)	Famsize	6.43	2.85
Training participation on coffee farm management (1 = yes; 0 = no)	Trainingpa	0.11	0.32
Social capital			
Membership of cooperatives	Mcoop	0.29	0.46
Fairtrade certification			
Farmer has fairtrade coffee certification (1 = yes; 0 = no)	Fairtrade	0.27	0.44

**Table 2:** Correlation coefficients for MVP adoption equation.

Equations	Coefficients (rho)	Stan. error	Z	*P* > *z*
Applying fertilizer vs renovating coffee tree stock	0.154	0.142	1.08	0.279
Soil conservation vs renovating coffee tree stock	0.074	0.165	0.45	0.654
Water management vs renovating coffee tree stock	0.485^*∗∗∗*^	0.119	4.07	0.000
Soil conservation vs applying fertilizer	0.422^*∗∗∗*^	0.135	3.12	0.002
Water management vs applying fertilizer	0.763^*∗∗∗*^	0.103	7.43	0.000
Water management vs soil conservation	0.717^*∗∗∗*^	0.108	6.62	0.000
Likelihood ratio test chi − square(6)=47.98^*∗∗∗*^				

*p* ^*∗∗∗*^ < 0.01.

**Table 3:** Coefficient estimates of the MVP model.

	Renovating coffee tree stocks				Fertilizer (organic and chemical) application			
Variables	Coefficients	Stand. error	Z	*P* > *Z*	Coefficients	Stand. error	Z	*P* > *Z*

Farsize (log)	0.228	0.392	0.58	0.561	−0.101	0.364	−0.28	0.781
Multplot	0.631	0.423	1.49	0.136	−0.833^*∗∗*^	0.391	−2.13	0.033
Remot (log)	−0.050	0.404	−0.12	0.901	−0.326	0.377	−0.87	0.386
Hiredlab	0.394	0.481	0.82	0.413	0.597	0.460	1.30	0.194
Exper (log)	0.736	0.549	1.34	0.180	0.984^*∗*^	0.578	1.70	0.089
Literacy	−1.182^*∗∗∗*^	0.301	−3.93	0.000	−0.387	0.265	−1.46	0.144
Famsize	0.118^*∗∗*^	0.050	2.34	0.019	0.062	0.043	1.44	0.149
Trainingpa	0.031	0.410	0.08	0.939	1.054^*∗∗*^	0.456	2.31	0.021
Mcoop	0.366	0.322	1.14	0.256	−0.210	0.379	−0.55	0.579
Fairtrade	0.630^*∗*^	0.367	1.72	0.086	−0.646	0.414	−1.56	0.119
Constant	−1.776	0.896	−1.98	0.047	−0.693	0.816	−0.85	0.396

	Implementing soil conservation measures				Practicing water management			
Variables	Coefficients	Standard error	Z	*P* > *Z*	Coefficients	Stand. error	Z	*P* > *Z*

Farsize (log)	−1.177^*∗∗∗*^	0.402	−2.93	0.003	0.153	0.410	0.37	0.709
Multplot	−0.232	0.485	−0.48	0.632	−0.504	0.419	−1.20	0.228
Remot (log)	0.644	0.463	1.39	0.165	−0.921^*∗∗∗*^	0.343	−2.69	0.007
Hiredlab	1.507^*∗∗∗*^	0.532	2.83	0.005	2.410^*∗∗∗*^	0.492	4.89	0.000
Exper (log)	0.804	0.633	1.27	0.204	−1.587^*∗∗∗*^	0.497	−3.19	0.001
Literacy	−0.082	0.298	−0.28	0.782	−1.379^*∗∗∗*^	0.275	−5.01	0.000
Famsize	0.027	0.048	0.56	0.573	−0.106^*∗∗∗*^	0.040	−2.68	0.007
Trainingpa	−0.132	0.590	−0.22	0.823	−0.608	0.547	−1.11	0.267
Mcoop	0.110	0.439	0.25	0.802	−0.275	0.332	−0.83	0.407
Fairtrade	−0.274	0.522	−0.53	0.600	0.594 ^*∗*^	0.354	1.68	0.093
Constant	−2.819	0.974	−2.89	0.004	4.196	0.831	5.05	0.000
Wald chi2(40) 134.96^*∗∗∗*^								
Log likelihood -281.74334								
Number of observations 153.000								

*p* ^*∗∗∗*^ < 0.01, *p*^*∗∗*^ < 0.05, and*p*^*∗*^ < 0.10.

**Table 4:** Ordered probit estimates of the factors influencing the number of SFEPs adoptions.

Variables	Coefficients	Standard error	Z. statistics	*P* > *z*
Fabrizio	−0.177	0.289	−0.61	0.541
Multplot	−0.345	0.320	−1.08	0.281
Remotnesslog	−0.234	0.293	−0.80	0.424
Hiredlab	1.569^*∗∗∗*^	0.396	3.96	0.000
Experlog	0.218	0.416	0.52	0.601
Literacy	−0.930^*∗∗∗*^	0.208	−4.47	0.000
Famsize	0.030	0.034	0.88	0.380
Trainingpa	0.194	0.336	0.58	0.563
Mcoop	−0.050	0.275	−0.18	0.857
Fairtrade	0.225	0.282	0.80	0.425
/cut1	−1.160	0.646		
/cut2	−0.195	0.635		
/cut3	0.662	0.641		
/cut4	1.256	0.654		
Number of observations = 153				
LR chi2(10) = 46.69 prob > chi2 < 0.001				
Log likelihood = −207.52725, pseudo *R*2 = 0.1011				

*p* ^*∗∗∗*^ < 0.01.

## Data Availability

The authors would like to declare that they can submit the data and datasets used for this study upon the publisher's request.

## Additional Points

This paper was not able to use a higher sample size because of financial budget limitation for data collection. As a result, a relatively higher sampling error (0.081) was used to decrease the number of sample respondents. Looking at the model presented here as an example, future studies should likely use small sampling error at 5% or less.
